# An Outcome-Oriented, Social–Ecological Framework for Assessing Protected Area Effectiveness

**DOI:** 10.1093/biosci/biab114

**Published:** 2021-11-03

**Authors:** Arash Ghoddousi, Jacqueline Loos, Tobias Kuemmerle

**Affiliations:** Humboldt-University Berlin, in Berlin, Germany; Leuphana University Lüneburg, in Lüneburg, Germany; Humboldt-University Berlin, in Berlin, Germany

**Keywords:** Aichi Target 11, area-based conservation, impact evaluation, national park, social–ecological systems

## Abstract

Both the number and the extent of protected areas have grown considerably in recent years, but evaluations of their effectiveness remain partial and are hard to compare across cases. To overcome this situation, first, we suggest reserving the term effectiveness solely for assessing protected area outcomes, to clearly distinguish this from management assessments (e.g., sound planning). Second, we propose a multidimensional conceptual framework, rooted in social–ecological theory, to assess effectiveness along three complementary dimensions: ecological outcomes (e.g., biodiversity), social outcomes (e.g., well-being), and social–ecological interactions (e.g., reduced human pressures). Effectiveness indicators can subsequently be evaluated against contextual and management elements (e.g., design and planning) to shed light on management performance (e.g., cost-effectiveness). We summarize steps to operationalize our framework to foster more holistic effectiveness assessments while improving comparability across protected areas. All of this can ensure that protected areas make real contributions toward conservation and sustainability goals.

Halting the ongoing loss of biodiversity is perhaps the greatest challenge humankind faces in the twenty-first century (Jones et al. 2018). Protected areas are a key tool in this context (Watson et al. 2014) and the global protected area network has recently expanded in major ways (around 11% and 211% increase in terrestrial and marine protected area coverages since 2010, respectively, corresponding to ca. 21.24 million square kilometers; Maxwell et al. 2020). However, maximizing protected area extent for its own sake is insufficient (Pressey et al. 2015, Pressey et al. 2017), and the conservation success of these expansions has been limited (Gill et al. 2017, Adams et al. 2019, Wolf et al. 2021). Many protected areas do not effectively safeguard biodiversity, and human pressures inside protected areas are sometimes as high as or even higher than in unprotected lands (Laurance et al. 2012, Geldmann et al. 2019). Ensuring conservation effectiveness therefore critically depends on better understanding the reasons why protected areas perform poorly in many situations, and this requires rigorous and evidence-based evaluation (Leverington et al. 2010, Watson et al. 2014, Ribas et al. 2020). Unfortunately, such evaluations are missing for most protected areas, and where effectiveness has been assessed, the evidence is often inconclusive (Zafra-Calvo and Geldmann 2020).

Rigorously assessing the effectiveness of protected areas has become more challenging as the goals of protected areas diversify from biodiversity conservation to economic, cultural, and development goals (Palomo et al. 2014, Watson et al. 2014, Pressey et al. 2015). This diverse set of goals for protected areas reflects a major shift in conservation paradigms in recent decades, from safeguarding wilderness and intact natural habitats toward promoting resilient social–ecological landscapes (Mace 2014). As a result, the phrase *protected area effectiveness* is nowadays used in manifold ways, including to assess whether protected areas safeguard biodiversity (Geldmann et al. 2013), whether protected areas reduce human pressures (Schulze et al. 2018, Geldmann et al. 2019), whether protected areas provide benefits to people (Naidoo et al. 2019), or whether protected areas are managed properly (Leverington et al. 2010, Coad et al. 2015). Such a diverse use of the term *effectiveness*, however, complicates or even inhibits cross-comparison and, therefore, more generalized insights (Rodrigues and Cazalis 2020). There is currently no clear, holistic definition of protected area effectiveness that would allow systematic effectiveness evaluations (Barnes et al. 2017, Maxwell et al. 2020).

Different strands of protected area evaluation have evolved largely in isolation from each other, resulting in diverging sets of indicators and methodologies (Eklund and Cabeza 2017). These typically focus on either ecological or social outcomes and often on individual indicators only (e.g., forest cover maintained, income to local people generated; for more examples see our literature review below). Such an approach risks conveying an oversimplified or even misleading picture of protected area effectiveness (Laurance et al. 2012, Watson et al. 2014, de Lange et al. 2016). For instance, protected areas with strict biodiversity protection goals often perform quite poorly on social outcomes (Oldekop et al. 2016, Zafra-Calvo et al. 2019), although they are not necessarily more effective in reducing human pressures either (Elleason et al. 2021). Likewise, forest cover is commonly used as a single ecological indicator of protected area effectiveness in broadscale comparative work (42% of the studies we reviewed; see below; Andam et al. 2008, Bowker et al. 2017, Wolf et al. 2021). However, around 40% of terrestrial protected areas worldwide have little to no tree cover (Digital Observatory for Protected Areas, https://dopa-explorer.jrc.ec.europa.eu), and even in protected areas with forests, tree cover has been proven to be a poor indicator of below-canopy biodiversity status (Burivalova et al. 2019, Green et al. 2020).

Recognizing the need for more holistic assessments of effectiveness, the multifaceted framework on protected area management effectiveness (PAME) has been developed and implemented widely across the world (Leverington et al. 2010, Coad et al. 2015). PAME assessments are based on information from management elements (e.g., design and planning), hypothesizing that improvements in these elements would foster positive conservation outcomes (Coad et al. 2015, Rodrigues and Cazalis 2020). However, these frameworks mainly focus on evaluating protected area management per se, such as the inflow of funding or a protected area's wider institutional settings. Yet, PAME insufficiently covers the diversity of protected area outcomes (e.g., largely disregarding human well-being or social equity; Stolton et al. 2019, Maxwell et al. 2020), and according to the typology of conservation evaluation approaches by Mascia and colleagues (2014), should be considered a management assessment rather than an impact evaluation tool. This difference could explain why the relationship between independently measured protected area outcomes and PAME scores can be weak or counterintuitive (Coad et al. 2015, Geldmann et al. 2018, Eklund et al. 2019). For example, a recent global meta-analysis shows that protected areas with sound management design and planning have a higher rate of people unsatisfied with decision-making processes in these protected areas (Zafra-Calvo and Geldmann 2020). Although they influence effectiveness, management factors are not by themselves indicators of protected area effectiveness; they need to be put into the context of protected area outcomes (e.g., biodiversity indicators).

In the present article, we argue for a clearer and more holistic definition of protected area effectiveness, that incorporates multiple protected area outcomes. First, we suggest that the phrase *protected area effectiveness* should be reserved exclusively for measuring a change in an outcome indicator affected by an individual protected area or a network of them, as it has been defined in impact evaluation approaches (Mascia et al. 2014, Pressey et al. 2015, Mascia et al. 2017). A robust evaluation of effectiveness should therefore measure the impact of protection on outcomes, which is best achieved using valid counterfactuals (i.e., comparable unprotected sites; Pressey et al. 2015). Such a clearer effectiveness definition will help to clarify the purpose of effectiveness assessments and help to disentangle contextual, mechanistic, and outcome variables (Eklund and Cabeza 2017). Second, we argue for a social–ecological perspective on protected areas (DeFries et al. 2010, Ban et al. 2013, Cumming and Allen 2017), because such a perspective can account for multiple dimensions of effectiveness (Meehan et al. 2020, Rodrigues and Cazalis 2020).

In the present article, we develop such an outcome-oriented, multidimensional, conceptual framework for a more holistic assessment of protected area effectiveness, rooted in social–ecological theory. In light of this framework, we review the scientific literature on protected area effectiveness assessments, and delineate key contextual and management elements (see below for definitions) for achieving effective protected areas. Finally, we provide examples of how available tools and data sets could be integrated under the umbrella of our outcome-oriented, social–ecological framework. We suggest that the adoption of our conceptual framework for assessing protected area effectiveness is timely for devising post-2020 global biodiversity targets that ensure the joint delivery of conservation and sustainability goals.

## A social–ecological framework for evaluating protected area effectiveness

The initial idea of protected areas was to maintain wilderness and to prohibit extraction of resources and other land uses by humans (West et al. 2006). This, partially forceful, segregation of humans and nature has since been criticized for its disengagement with human well-being, human rights and the United Nations’ Sustainable Development Goals (Brockington and Igoe 2006, Brockington et al. 2006, Menton et al. 2020). Moreover, the involvement of local stakeholders is now considered crucial for the legitimacy and the effectiveness of conservation (Andrade and Rhodes 2012, Oldekop et al. 2016). The fair integration of humans as beneficiaries is now widely agreed on, as is reflected in the Aichi Target 11 of the Convention of Biological Diversity (CBD). This recognition of humans both as custodians and victims of conservation interventions contributed to a paradigm shift that placed protected areas in relation to people and their activities rather than considering protected areas as isolated fragments of pristine nature (Mace 2014). A few recent studies have recognized this to jointly assess the social and ecological outcomes of protected areas (Oldekop et al. 2016, Ban et al. 2017, Burivalova et al. 2019). However, a wider framework to systematically recognize the range of protected area outcomes is so far missing.

**Figure 1.**
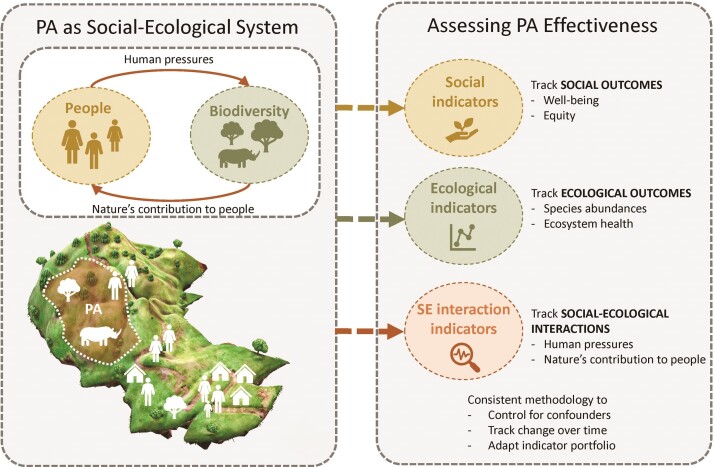
Following Ostrom (2009), we consider protected areas (PA) as embedded in social–ecological systems. Assessing their effectiveness should therefore consider three outcome-oriented dimensions: ecological outcomes, social outcomes, and social–ecological interactions.

A powerful way to consider interactions between people and nature in the context of protected areas is to view protected areas as embedded in wider social–ecological systems (DeFries et al. 2010, Palomo et al. 2014, Cumming et al. 2015). Social–ecological systems are characterized by distinct ecological and social subsystems, each with diverse components, as well as the complex interactions and feedbacks between these components (Ostrom 2009, Ban et al. 2013, Barnes et al. 2017). A social–ecological systems perspective pays particular attention to actors, how they acquire resources, how resource use feeds back on these actors, and how governance, such as change in use rights in a protected area, affects these interactions (Ostrom 2009, McGinnis and Ostrom 2014, Bodin 2017). Building on this theoretical framework, a social–ecological perspective should therefore help to design, structure, and implement more holistic and systematic assessments of protected area effectiveness (Ostrom 2007, Cumming and Allen 2017, Mascia et al. 2017). Importantly, such an assessment should occur across multiple outcome dimensions (i.e., factors in a social–ecological system that influence and are influenced by a management action; Meehan et al. 2020). In the present article, we propose protected area effectiveness assessments should address and be structured along three complementary dimensions (figure 1): ecological outcomes, social outcomes, and social–ecological interactions. Any of these dimensions can (and often should) entail multiple indicators, thereby yielding a multifaceted assessment of protected area effectiveness. Below, we provide furthermore, explanation on each of these three dimensions.

### Ecological outcomes

Ecological outcomes refer to biodiversity targets (e.g., species or ecosystems of conservation concern), such as their abundance, condition, or functioning (Gray et al. 2016, Barnes et al. 2017). Moreover, protected areas may have targets that benefit biodiversity indirectly, such as fostering connectivity or supporting viable metapopulations (Pressey et al. 2015). There is currently mixed evidence on the global effectiveness of protected areas in delivering ecological outcomes, depending on the indicators used. For instance, although protected areas appear to be effective in reducing forest loss (Geldmann et al. 2013, Burivalova et al. 2019, Wolf et al. 2021), the evidence for protected areas safeguarding viable populations of species of conservation concern is limited and inconclusive (Craigie et al. 2010, Gray et al. 2016). It is therefore critically important to move beyond simplified, single-indicator assessments to better capture the complexity of biodiversity across taxa, facets, and hierarchical organization (from genetic diversity to ecosystems).

We emphasize that any assessment of the ecological outcomes of protected areas will depend on the quality of available biodiversity data (Geldmann et al. 2013), and the most powerful assessments will be based on long-term monitoring data—requiring major and continuous investments (Pereira et al. 2013). Scarce or missing biodiversity data, especially from outside protected areas or from the time before their establishment, can preclude comprehensive assessments of ecological outcomes (Pereira et al. 2013). Likewise, data limitations can prohibit the use of the strongest methods to quantify the impact (Coad et al. 2015, Pressey et al. 2015, Eklund and Cabeza 2017). However, major advances in systematically monitoring even large and inaccessible landscapes via satellites and remote sensors (e.g., through essential biodiversity variables), crowd-sourced information, or more efficient field methods such as eDNA are rapidly opening up new opportunities for improved and even retrospective assessments (Pereira et al. 2013, Joppa et al. 2016, Skidmore et al. 2021).

### Social outcomes

Social outcomes in our conceptual framework refer to two categories of social indicators: the well-being of people directly affected by the protected area and equity, because various members of the community may be affected differently by protected area interventions. Such social outcomes have only recently gained major attention in effectiveness evaluations (Zafra-Calvo et al. 2017, Naidoo et al. 2019) but are typically assessed separately from ecological outcomes (but see Ban et al. 2017, Burivalova et al. 2019 and Oldekop et al. 2016). This limits our ability to cross-reference between these dimensions.

Many protected areas explicitly seek to improve the well-being of local communities and Indigenous Peoples (Corrigan et al. 2018) and some effectiveness evaluations, such as the Social Assessment of Protected Areas (SAPA) tool, focus particularly on these outcomes (Moreaux et al. 2018). When tracking progress in human well-being, indicators such as living standards (e.g., income, employment, health), education, and environmental and security issues are commonly used (de Lange et al. 2016, Corrigan et al. 2018). Although most evaluations focus on the material aspects or distribution of monetary benefits of protected areas among local people (de Lange et al. 2016), a more nuanced understanding of well-being would also include relational (e.g., social interactions) and subjective (e.g., identity, cultural values) aspects. As with biodiversity data, there is a general lack of time-series data on human well-being (de Lange et al. 2016), and existing data is often coarse in scale. A diverse and transparent set of well-being indicators that can be efficiently monitored is needed, as well as recognition of the value of gathering such data among funding agencies and government bodies.

Aichi Target 11 urges signatories to manage protected areas equitably, acknowledging that the costs of protected areas are often primarily borne by certain groups (e.g., local people). Although mitigating inequity is an inherent goal within sustainability, it is also an instrumental consideration in the conservation context, because inequity may undermine the goals of protected areas (Klein et al. 2015). However, obtaining data on equity is complex, because it may be perceived differently by various stakeholders (Klein et al. 2015, Moreaux et al. 2018). Existing tools focusing on social outcomes either do not fully address equity (e.g., SAPA, PAME, and the IUCN Green List for Protected and Conserved Areas), or are too costly to be implemented (e.g., IUCN Best Practice Guidelines of Governance of Protected Areas; Moreaux et al. 2018, Zafra-Calvo et al. 2019). The application of the environmental justice framework (Schlosberg 2007) would help to assess equity aspects of protected areas with regard to distribution (e.g., the distribution of protected area costs and benefits), procedure (e.g., decision-making processes), and recognition (e.g., valuing social and cultural diversity and plurality; Schreckenberg et al. 2016, Zafra-Calvo et al. 2017). Interestingly, a global assessment based on these aspects shows contrasting trends in how protected areas progress toward social equity, with significant inadequacies in decision-making, transparency, dispute resolution mechanisms, and the recognition of the local people's rights (Zafra-Calvo et al. 2019). This furthermore, emphasizes the need to consider equity outcomes alongside well-being in assessments of protected area effectiveness.

### Social–ecological interactions

For the purpose of our framework, we follow Soga and Gaston (2020) to define *social–ecological interactions* as the “direct interactions between individual people and nature.” Indicators of social–ecological interactions should therefore capture the dynamic interactions and feedbacks between the social and ecological subsystems (Barnes et al. 2017, Eklund and Cabeza 2017). For example, poaching as a social–ecological interaction involves the removal of wildlife from an environment and provides poachers with nutritional and/or monetary benefits. This interaction may affect a range of protected area outcomes, such as ecological (e.g., the loss of key ecological functions where large mammals are hunted out), social outcomes (e.g., livelihood improvements because of higher income if bushmeat is sold) or other social–ecological interactions (e.g., reduced opportunities for wildlife tourism), highlighting the importance of a multidimensional approach for evaluating the impacts of protected areas. We suggest two categories of interactions are particularly relevant for evaluating protected area effectiveness: human pressures on biodiversity targets in protected areas and nature's contribution to people (NCP) associated with protected areas, including negative and positive contributions (Díaz et al. 2018).

About one-third of protected areas worldwide are facing intense human pressures (Jones et al. 2018). A key to protected area effectiveness is therefore the mitigation of these pressures, such as habitat destruction and overexploitation (Laurance et al. 2012, Pressey et al. 2015, Schulze et al. 2018). Global studies of human pressures in protected areas increasingly use broad, remotely sensed indicators (e.g., fire incidence; Nelson and Chomitz 2011) or compound human pressure surrogates (e.g., combining indicators such as human population density or nighttime lights; Jones et al. 2018, Geldmann et al. 2019). However, such indicators do not always reflect well pressures on biodiversity, and in some cases, human activities captured by these indicators may even be in line with biodiversity conservation goals (e.g., pasture management to maintain seminatural areas; Gavin et al. 2018). Therefore, to estimate a true representation of human pressures that negatively affect species and ecosystems, it is essential to consider the drivers of biodiversity loss (e.g., land-use change, poaching) at a local scale. Data for many human pressures on terrestrial protected areas are increasingly available, at finer resolution and higher quality than in the past (Joppa et al. 2016, Schulze et al. 2018). However, some of these data sets remain uncertain and major gaps still prevail, particularly at regional to local scales (Mammides 2020). For example, apart from forest loss, our understanding of vegetation changes for most ecosystems is incomplete (Joppa et al. 2016), and data on the spatial footprint and intensity of key land-use practices (e.g., livestock grazing) or overexploitation patterns (e.g., poaching) remain very patchy (Gavin et al. 2010, Kuemmerle et al. 2013). As with biodiversity data, there is a considerable promise that this will change soon, given recent advances in remote sensing, crowd-sourced data collection, and their integration with on-the-ground data on human pressures (e.g., via the Spatial Monitoring and Reporting Tool).

Our second major category in terms of social–ecological interactions is NCP (Díaz et al. 2018), which, in our case, refers to the positive (e.g., food provision, water purification), as well as negative (e.g., diseases, livestock loss to predators) contributions of protected areas to people's quality of life. NCP evolved from broadening the ecosystem services concept to better incorporate social science perspectives, different notions of culture, and local and Indigenous knowledge (Díaz et al. 2018). Although the NCP concept has been criticized for a lack of novelty (Braat 2018), NCP is the current terminology used by the Intergovernmental Science-Policy Platform on Biodiversity and Ecosystem Services (IPBES 2019) and we, therefore, used it in the present article too. Importantly, in the context of effectiveness evaluations, NCP should not be confused with the social outcomes of protected areas, such as human well-being (which may be underpinned by NCP; Reyers et al. 2013). NCP covers monetary (e.g., production of biomass-based fuels), nonmonetary (e.g., provision of opportunities for religious or spiritual experiences), and regulatory (e.g., regulation of air quality) aspects (Díaz et al. 2018). Although many tools and approaches for assessing NCP have been proposed (e.g., measuring biological carbon storage and sequestration, crop pollination), indicators derived from such assessments have rarely been included in protected area effectiveness evaluations. Encouragingly, the new IUCN Protected Area Benefits Assessment Tool+ (PA-BAT+) aims at collating and assessing information on local stakeholders’ perceptions on the benefits of protected areas (Ivanić et al. 2020), which could complement quantitative approaches in assessing NCP. However, following the NCP concept, PA-BAT+ should furthermore, expand to also entail the negative impacts of protected areas on local stakeholders. As the conceptual basis and tools for tracking NCP are maturing, it will become possible to include NCP indicators in protected area effectiveness assessments (Maxwell et al. 2020). This will help us to better understand the interlinks between positive and negative NCP and other protected area outcomes.

## Protected area effectiveness literature

To better understand the nature of protected area effectiveness assessments documented in the literature to date, and to evaluate how these assessments covered the three dimensions of our proposed framework, we carried out a comprehensive literature review. To identify studies, we searched for the terms “protected area* effectiveness,” “national park* effectiveness,” “biosphere reserve* effectiveness,” “nature reserve* effectiveness,” “effectiveness of protected area*,” “effectiveness of national park*,” “effectiveness of biosphere reserve*,” “effectiveness of nature reserve*,” “protected area* impact*,” “national park* impact*,” “biosphere reserve* impact*,” “nature reserve* impact*,” “impact* of protected area*,” “impact* of national park*,” “impact* of biosphere reserve*,” and “impact* of nature reserve*” in the title, keywords, or abstracts of peer-reviewed articles and review papers published until 2021 in English as they were indexed in the Scopus database (www.scopus.com). We acknowledge that many protected area effectiveness assessments are not published as scientific articles or that there are additional studies in other languages than English. Although our literature search did not capture all past work on protected area effectiveness, our informative and indicative sample of 366 papers demonstrates how the effectiveness terminology has been used to date in the scientific literature. We further narrowed down our search to terrestrial protected areas and removed those papers related to marine and freshwater protected areas (*n* = 36) to achieve a set of studies with potentially comparable effectiveness indicators. We do, however, acknowledge the marked advances in assessing marine protected area effectiveness in recent years (see Gill et al. 2017 and Meehan et al. 2020 for reviews). Three independent reviewers screened the abstracts of the remaining papers to determine whether the study is directly relevant to the assessment of protected area effectiveness (e.g., not a gap analysis) and fitting the definition of impact evaluation by Mascia and colleagues (2014). The reviewers cross-checked their classifications and referred to the full text when there was a disagreement in classifications. The final list contained 150 papers fitting our criteria (see supplemental table S1). For each of them, we indicated the dimensions of effectiveness covered, assessed according to our conceptual framework (social, ecological, or social–ecological interactions outcomes), and their indicators. We followed the PRISMA protocol in reporting our literature review steps (see supplemental figure S1).

**Figure 2.**
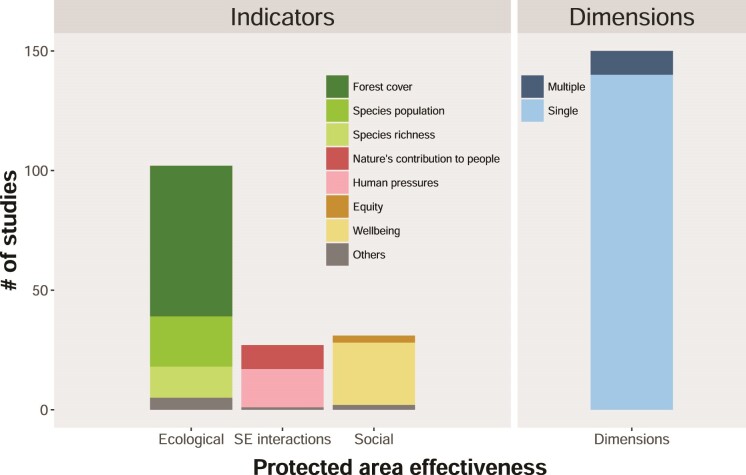
The use of different protected area effectiveness outcome indicators (left) and dimensions (right) in 150 reviewed studies. Abbreviation: SE interactions, social–ecological interactions.

In the final list of papers, only nine studies (table 1) had measured more than one dimension of protected area outcomes, whereas the remaining 141 studies assessed one outcome dimension only (figure 2). The most frequently measured outcome dimension was the ecological one (68% of the studies; figure 2) reflecting the primary goal of protected areas and the continued strong focus on this dimension. Forest cover was the most commonly used indicator of protected area effectiveness (42% of the studies). The remaining studies assessed social–ecological interactions (18%), and social outcomes indicators (21%), illustrating that social and social–ecological interactions outcomes continue to be neglected in protected area effectiveness assessments.

**Table 1.** The few studies (6%) of protected area effectiveness assessment that assessed more than one outcome dimension still assess effectiveness only partially.

Dimensions	Ecological		Social		Social–ecological interactions	
Examples of indicators	Habitat extent, quality	Species abundance, diversity	Human well-being	Social equity	Human pressures	Nature's contribution to people
Bolivia (all protected areas)^a^	Forest cover	–	Poverty index	–	–	–
Cambodia (Kulen Promtep Wildlife Sanctuary and Preah Vihear Protected Forest)^b^	–	–	Poverty (Basic Necessities Survey)	–	–	Nontimber forest products and agricultural productivity
Chile (all protected areas)^c^	Plant productivity	Biodiversity (species richness)	–	–	–	Carbon storage (vegetation biomass and soil organic carbon) and agricultural production (gross production)
Colombia (protected areas in one district)^d^	Habitat quality (natural land covers) and ecological systems (representation, rarity, remanence and rate of loss)	Sensitive species richness (endemic, migratory and endangered species)	–	–	–	Scenic beauty, water provision
Costa Rica (all protected areas)^e^	Forest cover	–	Poverty index	–	–	–
Costa Rica, Indonesia and Thailand (forested protected areas)^f^	–	–	Poverty	–	–	Carbon storage
Ghana (Mole National Park) and Tanzania (Tarangire National Park)^g^	–	–	Employment, cultural values, access to forest products and land	Equity in economic benefits, communication with the community, inclusion in park governance	–	Tourism, wildlife depredation
Global (19,486 protected areas)^h^	Forest cover	–	–	–	Human footprint index	–
Tanzania (East Usambara Mountain)^i^	Forest cover and connectivity	Exotic and native species	Loss of livelihood	–	–	–
UK (Northumberland National Park)^j^	–	–	Income, rural development	–	–	Tourism

a Hanauer and Canavire-Bacarreza (2015). ^b^Clements and colleagues (2014). ^c^Durán and colleagues (2013). ^d^Gárcia Márquez and colleagues (2017). ^e^Ferraro and Hanauer (2011). ^f^Ferraro and colleagues (2015). ^g^Abukari and Mwalyosi (2020). ^h^Elleason and colleagues (2021). ^i^Hall and colleagues (2014). ^j^Gandariasbeitia (2010).

## Key elements for achieving protected area effectiveness

Achieving the range of social, ecological and social–ecological outcomes outlined above depends on management and contextual elements (Rodrigues and Cazalis 2020). Management elements reflect decisions about the actual management of protected areas (e.g., staff, budget). Contextual elements reflect decisions taken at the time of the establishment of protected areas (e.g., size, remoteness). These elements are assumed to provide the essential means for protected areas in achieving their biodiversity and socioeconomic goals or in enhancing their resilience (Coad et al. 2015, Rodrigues and Cazalis 2020). Therefore, evaluating the link between these elements and protected area outcomes is useful for detecting strengths and shortfalls in protected areas, as well as for finding ways to improve their effectiveness (Gill et al. 2017, Coad et al. 2019).

A range of indicators from the management cycle (i.e., planning, inputs, processes, outputs and outcomes) is typically used to assess protected area management. These indicators can broadly be grouped into design and planning, capacity and resources, monitoring and enforcement systems, and decision-making arrangements (Geldmann et al. 2018). Many of these indicators have been evaluated within PAME assessments over 27,600 times in 177 countries, using 57 different methods that normally involve questionnaires completed by protected area managers and other stakeholders (https://pame.protectedplanet.net). A recent study (Coad et al. 2019) shows that according to PAME assessments less than a quarter of protected areas have adequate resources in terms of staffing and budget. PAME assessments, therefore, could provide insights into how protected areas operate and under which conditions they could be effective. This is particularly important as the relationship between management inputs and protected area outcomes is complex (Pressey et al. 2017). Recent assessments also clearly show that management interventions are necessary, but they are not sufficient to ensure protected area effectiveness (Coad et al. 2015, Geldmann et al. 2018, Eklund et al. 2019).

Contextual elements include factors such as protected area design (e.g., size, shape), broader socioeconomic conditions (e.g., different levels of economic growth, corruption, legislative framework) and governance (e.g., strict protection versus multiple-use landscape), all of which are known to influence protected area outcomes (Bowker et al. 2017, Graham et al. 2021, Wolf et al. 2021). Importantly, the objectives of protected areas, commonly reflected in their IUCN categories (categories I–VI), vary and determine the focus of protected areas on certain outcomes, which should be considered in impact evaluations (Pressey et al. 2015, Elleason et al. 2021). This is particularly important given the increasing spatial coverage of other effective area-based conservation measures, such as private or community-based reserves (Maxwell et al. 2020, Palfrey et al. 2021), which typically have a broader suite of objectives than biodiversity conservation alone.

Assessing protected area effectiveness in terms of ecological, social, and social–ecological outcomes, as we suggest in the present article, provides opportunities to better understand how the management and contextual elements relate to effectiveness (Schoon et al. 2021). Systematically tracking diverse management indicators, such as funding inflows, interventions carried out, or labor input, would allow the comparison of management indicators with independently measured effectiveness indicators. This would allow the derivation of “second-order” effectiveness measures that relate inputs (e.g., funding, labor) to protected area outcomes (e.g., ecological or well-being), in order to identify which measures are most cost-effective; a key concern of conservation organizations and funders. Importantly, data collection on protected area management should be conducted using standard protocols, and ideally in a participatory manner to avoid subjective assessments (Eklund and Cabeza 2017, Moreaux et al. 2018, Stolton et al. 2019). Likewise, original data on indicators should be made available, because simplifying them into a single compound score can obscure the main strengths and deficiencies of a specific protected area and complicate cross-comparisons (Coad et al. 2015, Stolton et al. 2019). Ideally, protected area management evaluations (such as PAME) should be carried out independently from protected area outcome assessments (such as the framework proposed in the present article), but they can be presented on a common platform to facilitate robust and concurrent assessments of protected area inputs and outcomes.

## Operationalization of the framework

Embracing protected areas as embedded in wider social–ecological systems requires acknowledging the diversity and complexity of these systems (Ban et al. 2013, Cumming and Allen 2017). Indeed, ignoring this complexity is a key reason for oversimplified protected area assessments. Our framework can represent a key step toward remedying this situation, by systematically structuring complexity (e.g., assessing effectiveness along the three dimensions proposed) and by clarifying terminology (e.g., separating assessments of protected area outcomes from management performance). Both are critically important for enabling comparative analyses across individual effectiveness assessments and, therefore, for identifying high-level combinations of drivers and outcomes of protected area performance (Schoon et al. 2021) and, more generally, for knowledge cocreation (van Riper et al. 2016, Magliocca et al. 2018, Meyfroidt et al. 2018).

Operationalizing our framework requires applying and adjusting it to a specific social–ecological context while retaining its internal structure (e.g., assessing protected area outcomes along three dimensions). Our framework is rooted in social–ecological theory, and therefore, guidelines on how to operationalize research on social–ecological systems generally (Binder et al. 2013, Leslie et al. 2015, Biggs et al. 2021) and specifically for protected areas (Cumming et al. 2015, Ban et al. 2017, Martín-López et al. 2017) provide highly useful conceptual and methodological advice on how to adopt this framework for a specific case study. Our goal in the present article is not to reproduce these guidelines but to highlight general steps to consider when operationalizing our framework:

### Define system boundaries

Determining the boundaries of the system is a fundamental consideration for the assessment of social–ecological systems (Biggs et al. 2021). In the case of protected area evaluation, a system could be composed of a single protected area or a network of them and their surroundings. Defining the system should include identifying and mapping core ecological units (e.g., watershed, ecosystems), as well as key social variables (e.g., land use, demography). These can then be integrated to identify social–ecological boundaries (e.g., a zone of interaction; DeFries et al. 2010). System boundaries can be validated using participatory approaches (Martín-López et al. 2017).

### Identifying appropriate indicators

The selection of indicators for tracking protected area impacts across our three dimensions needs to consider the given social–ecological context (e.g., select the most relevant indicators), as well as practicality aspects (e.g., select indicators that can be monitored cost-effectively; Leslie et al. 2015). Indicators must reliably reflect the status and trends in relevant outcomes and should respond to conservation interventions (Jones et al. 2011, Pressey et al. 2015). Although remotely sensed indicators can be valuable resources, the holistic effectiveness assessment we advocate for in the present article will ideally integrate both ground monitoring data and remotely sensed indicators (Mascia et al. 2014). Because of methodological advances, detailed time-series ecological and social indicators are increasingly available (Pereira et al. 2013, Joppa et al. 2016), including on protected area management interventions (Geldmann et al. 2018, Stolton et al. 2019). The selection of a relevant and feasible set of indicators is best done using a transdisciplinary, participatory process, involving protected area managers (Ban et al. 2017).

### Gather relevant data

Assessing conservation impact requires data on indicators both from inside protected areas, as well as from comparable unprotected counterfactual sites (Ferraro 2009). The strongest impact assessments become possible where consistent time series of indicators are collected, requiring longer-term investments in data collection and curation (Pressey et al. 2015). Furthermore, some outcome indicators might be uncertain proxies for protected area impact (e.g., changes in human pressures decoupled from conservation interventions) or might just be difficult to collect outside protected areas at present (e.g., state of biodiversity). In such situations, regular reevaluation of indicator choice, data collection methodologies and resource allocation in an adaptive process can help to gradually move closer to indicators that inform both research and management.

### Assess impact

Effectiveness assessments are most powerful when solid experimental designs and appropriate analytical tools are used to evaluate impact (Mascia et al. 2014, Butsic et al. 2017, Ribas et al. 2020). Care must be taken to properly account for selection bias in protected area placement (e.g., the *high and far* bias; Joppa and Pfaff 2009). This includes choosing valid counterfactuals (see above), as well as considering selection bias when interpreting the magnitude of protected area impact (Pressey et al. 2015, Eklund and Cabeza 2017). Moreover, when time-series indicators are available, robust before-after-control assessments are possible (Christie et al. 2019).

### Comparison across sites and scales

Our framework structures effectiveness assessments, while allowing to consider diverse social–ecological contexts, thereby fostering cross-site comparisons (Ban et al. 2013). Importantly, synthesis efforts can in principle focus on subsets of our framework (e.g., case studies assessing a specific dimension of effectiveness) or more holistic notions of effectiveness as targeted by our framework as a whole. A range of methods for knowledge generalization is now available for this purpose, including more quantitative (e.g., system meta-analyses) or qualitative approaches (e.g., archetype identification; Ban et al. 2017, Magliocca et al. 2018).

Although operationalizing our framework for a given case study will be an effort, the nine multidimensional studies identified in our review (table 1) provide practice examples on how this has already partially been achieved. Although our systematic approach on identifying these studies stems from an academic perspective, we consider the reconciliation of effectiveness evaluations as an opportunity to collaboratively include different, place-specific values and perspectives of various protected area stakeholders, managers and researchers (Apostolopoulou et al. 2021). Specifically, these case studies exemplify effectiveness evaluations that go beyond single dimensions and indicators, that compared across different protected area systems (e.g., sites or networks), that evaluated diverse goals (e.g., strict protected or multiple use) and that assessed different governance types (e.g., community based or governmental).

## Conclusions

Assessing the effectiveness of protected areas is increasingly important in conservation science and practice. In the present article, we argue that a social–ecological perspective can provide a more holistic and systematic framing for effectiveness assessments. We suggest that protected area evaluations should more clearly distinguish between assessments of protected area effectiveness, which focus on measuring the impact of protected areas on diverse outcomes, and evaluations of the management of protected areas. In other words, well-managed protected areas could still be ineffective in maintaining key biodiversity features, whereas poorly managed protected areas could be effective in reaching their conservation goals. A social–ecological framework and an emphasis on outcomes can more clearly separate between these two aspects. Such an approach is also an opportunity to move beyond the current plethora of often inconclusive protected area assessments, whose ambiguity may at least in part be because of a diversity of framings, approaches, and indicators used, as well as the inconsistent use of the term *effectiveness*. Streamlining the effectiveness terminology and consolidating existing databases relevant to protected area evaluation more clearly into outcome dimensions and management indicators would be a useful next step in this regard.

Scaling up knowledge about protected area effectiveness across sites requires a common framework allowing for cross-comparison. Our framework enables this; however, it does not resolve the trade-off between generality and specificity inherent to social–ecological research (Sitas et al. 2019). A common framework can structure assessments while retaining social–ecological nuance, therefore ensuring appropriate levels of abstraction for comparisons can be identified (i.e., similar to other frameworks in social–ecological research such as the ecosystem services framework; Millennium Ecosystem Assessment 2005). As our literature review shows, protected area effectiveness assessments have so far largely ignored the generality-specificity trade-off, by typically using single effectiveness indicators, by using mainly ecological indicators, or by conveying false notions of comparability through using similar terminology where it is not justified.

Consistently connecting top-down initiatives such as the World Database on Protected Areas, the IUCN Green List for Protected and Conserved Areas, SAPA, PA-BAT+, and the Global Database on PAME would provide major opportunities toward reaching a comprehensive and transparent platform for cross-comparison of protected area effectiveness. As broadscale, global indicators and data sets have limitations (Laurance et al. 2012, Watson et al. 2014, de Lange et al. 2016), site-level, locally grounded data will often be more powerful for understanding protected area impacts, management inputs, and the cost-effectiveness of interventions. Governmental agencies and donors should, therefore, support the consistent collection of site-level data on protected area outcomes and management interventions.

The recent increase in the area coverage of protected areas is a major conservation success. Ensuring the effectiveness of these protected areas should now become a top priority for the coming decades, particularly considering that protected area effectiveness overall is still questionable (Gill et al. 2017, Adams et al. 2019, Maxwell et al. 2020). We suggest that an outcome-oriented, social–ecological framework of protected area effectiveness would be highly beneficial to measure progress toward the post-2020 CBD targets. This could provide critical information on when and how protected areas are effective, ultimately helping to identify the best area-based conservation approaches to conserve species and habitats and improve human well-being.

## Supplementary Material

biab114_Supplemental_File
