# Supplementary material for: An Outcome-Oriented, Social–Ecological Framework for Assessing Protected Area Effectiveness
Source: Bioscience. 2021 Nov 3;72(2):201–12. doi: 10.1093/biosci/biab114 (PMC8824764; doi:10.1093/biosci/biab114)
Supplement: biab114_Supplemental_File [file biab114_supplemental_file.docx]

**An Outcome-oriented, Social-ecological Framework for Assessing Protected Area Effectiveness**

Arash Ghoddousi^1,*^, Jacqueline Loos^2,3^, and Tobias Kuemmerle^1,4^

^1^ Geography Department, Humboldt-University Berlin, Unter den Linden 6, 10099 Berlin, Germany

^2^ Institute of Ecology, Leuphana University Lüneburg, Universitätsallee 1, 21335 Lüneburg, Germany

^3^ Social-Ecological Systems Institute, Leuphana University Lüneburg, Universitätsallee 1, 21335 Lüneburg, Germany

^4^ Integrative Research Institute on Transformation in Human-Environment Systems, Humboldt-University Berlin, Unter den Linden 6, 10099 Berlin, Germany

* Corresponding author: Arash Ghoddousi, Geography Department, Humboldt-University Berlin, Unter den Linden 6, 10099 Berlin, Germany; +49-30-2093-5394; [arash.ghoddousi@hu-berlin.de](mailto:arash.ghoddousi@hu-berlin.de)

**Supplementary Materials**

**
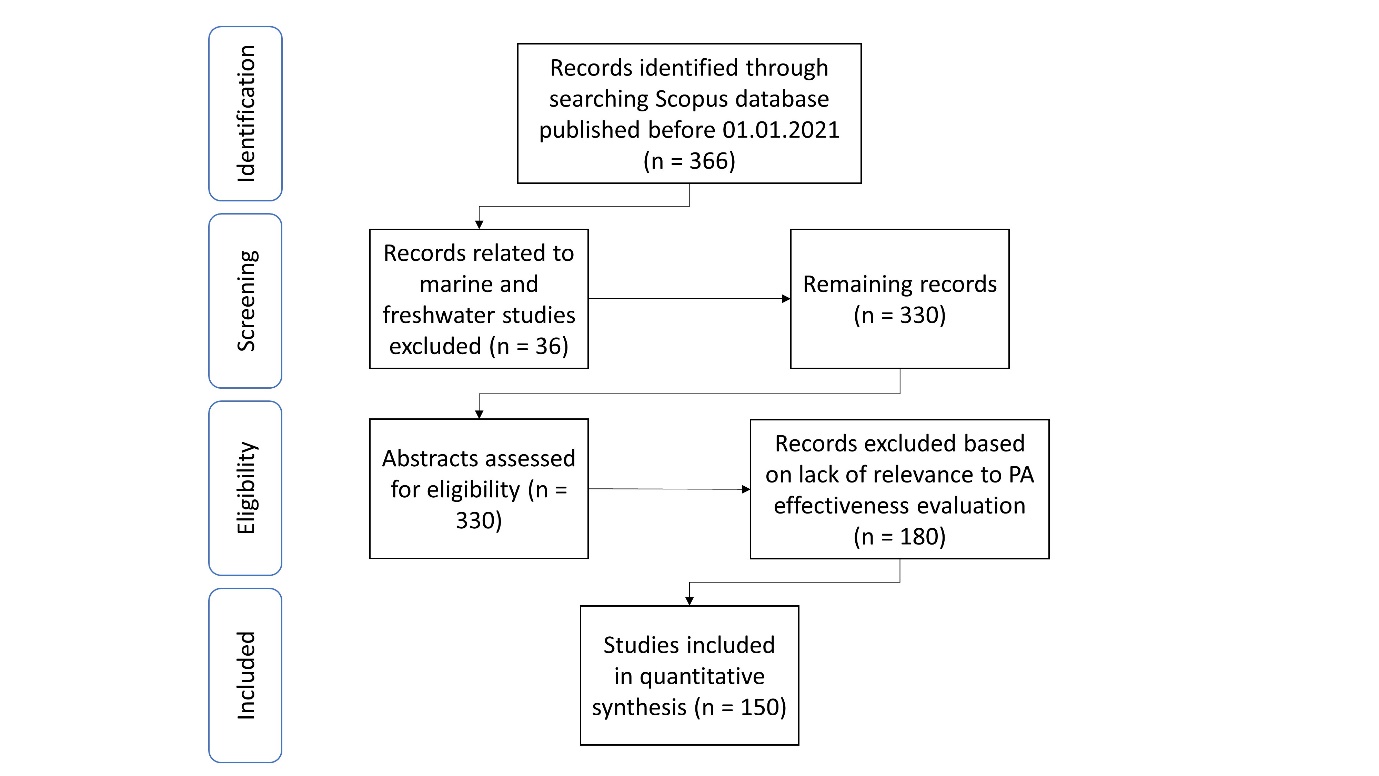
**

Figure S1. Flowchart outlining different steps in literature search following Preferred Reporting Items for Systematic Reviews and Meta-Analysis (PRISMA) protocol.

Table S1. Table of 150 studies considered as terrestrial protected area effectiveness assessment from our targeted literature review using Scopus data base (SE interactions: social-ecological interaction).

| # | Authors | Title | Year | Source title | Dimension | Indicator |
| --- | --- | --- | --- | --- | --- | --- |
| 1 | Kiffner C., Binzen G., Cunningham L., Jones M., Spruiell F., Kioko J. | Wildlife population trends as indicators of protected area effectiveness in northern Tanzania | 2020 | Ecological Indicators | Ecological | Species population |
| 2 | Kearney S.G., Adams V.M., Fuller R.A., Possingham H.P., Watson J.E.M. | Estimating the benefit of well-managed protected areas for threatened species conservation | 2020 | ORYX | SE interactions | Human pressures |
| 3 | Ortiz D.A., Dueñas J.F., Villamarín F., Ron S.R. | Long-Term Monitoring Reveals Population Decline of Spectacled Caimans (*Caiman crocodilus*) at a Black-Water Lake in Ecuadorian Amazon | 2020 | Journal of Herpetology | Ecological | Species population |
| 4 | Ayivor J.S., Gordon C., Tobin G.A., Ntiamoa-Baidu Y. | Evaluation of management effectiveness of protected areas in the Volta Basin, Ghana: perspectives on the methodology for evaluation, protected area financing and community participation | 2020 | Journal of Environmental Policy and Planning | SE interactions | Human pressures |
| 5 | Heringer G., Almeida T.E., Dittrich V.A.D.O., Salino A. | Assessing the effectiveness of protected areas for the conservation of ferns and lycophytes in the Brazilian state of Minas Gerais | 2020 | Journal for Nature Conservation | Ecological | Species richness |
| 6 | Maria Restello R., Battistoni D., Renan Sobczak J., Teresa Valduga A., Balvedi Zackrzevski S.B., Maria Zanin E., Secretti Decian V., Ubiratan Hepp L. | Effectiveness of protected areas for the conservation of aquatic invertebrates: A study-case in southern brazil [Efetividade das áreas protegidas na conservação de invertebrados aquáticos: Um estudo de caso no Sul do Brasil] | 2020 | Acta Limnologica Brasiliensia | Ecological | Species richness |
| 7 | Souza A.C.D., Prevedello J.A. | The importance of protected areas for overexploited plants: Evidence from a biodiversity hotspot | 2020 | Biological Conservation | Ecological | Species population |
| 8 | Wen Z., Cai T., Feijó A., Xia L., Cheng J., Ge D., Yang Q. | Using completeness and defaunation indices to understand nature reserve's key attributes in preserving medium- and large-bodied mammals | 2020 | Biological Conservation | Ecological | Species population |
| 9 | Xu X., Jiang B., Chen M., Bai Y., Yang G. | Strengthening the effectiveness of nature reserves in representing ecosystem services: The Yangtze River Economic Belt in China | 2020 | Land Use Policy | SE interactions | Nature's contribution to People |
| 10 | Yoh N., Azhar I., Fitzgerald K.V., Yu R., Smith-Butler T., Mahyudin A., Kingston T. | Bat ensembles differ in response to use zones in a tropical biosphere reserve | 2020 | Diversity | Ecological | Species population |
| 11 | Negret P.J., Marco M.D., Sonter L.J., Rhodes J., Possingham H.P., Maron M. | Effects of spatial autocorrelation and sampling design on estimates of protected area effectiveness | 2020 | Conservation Biology | Ecological | Forest cover |
| 12 | Cazalis V., Princé K., Mihoub J.-B., Kelly J., Butchart S.H.M., Rodrigues A.S.L. | Effectiveness of protected areas in conserving tropical forest birds | 2020 | Nature Communications | Ecological | Species richness |
| 13 | Ford S.A., Jepsen M.R., Kingston N., Lewis E., Brooks T.M., MacSharry B., Mertz O. | Deforestation leakage undermines conservation value of tropical and subtropical forest protected areas | 2020 | Global Ecology and Biogeography | Ecological | Forest cover |
| 14 | Ferreira G.B., Collen B., Newbold T., Oliveira M.J.R., Pinheiro M.S., de Pinho F.F., Rowcliffe M., Carbone C. | Strict protected areas are essential for the conservation of larger and threatened mammals in a priority region of the Brazilian Cerrado | 2020 | Biological Conservation | Ecological | Species richness |
| 15 | Maldonado A.D., Valdivielso A., Rescia A., Aguilera P.A. | Probabilistic graphical models for species richness prediction: Are current protected areas effective to face climate emergency? | 2020 | Global Ecology and Conservation | Ecological | Species richness |
| 16 | Gu C., Zhao P., Chen Q., Li S., Li L., Liu L., Zhang Y. | Forest cover change and the effectiveness of protected areas in the Himalaya since 1998 | 2020 | Sustainability (Switzerland) | Ecological | Forest cover |
| 17 | Yin L., Dai E., Zheng D., Wang Y., Ma L., Tong M. | Spatio-temporal analysis of the human footprint in the Hengduan Mountain region: Assessing the effectiveness of nature reserves in reducing human impacts | 2020 | Journal of Geographical Sciences | SE interactions | Human pressures |
| 18 | Elleason M., Guan Z., Deng Y., Jiang A., Goodale E., Mammides C. | Strictly protected areas are not necessarily more effective than areas in which multiple human uses are permitted | 2020 | Ambio | Ecological & SE interactions | Forest cover & human pressures |
| 19 | Jones N., Malesios C., Kantartzis A., Dimitrakopoulos P.G. | The role of location and social impacts of protected areas on subjective wellbeing | 2020 | Environmental Research Letters | Social | Human wellbeing |
| 20 | Mojo D., Oduor A.M.O., Fu C., Bai Y., Long H., Wang G., Zhang L. | Effects of protected areas on welfare of local households: The case of Maasai Mara National Reserve in Kenya | 2020 | People and Nature | Social | Human wellbeing |
| 21 | Abukari H., Mwalyosi R.B. | Local communities’ perceptions about the impact of protected areas on livelihoods and community development | 2020 | Global Ecology and Conservation | Social & SE interactions | Human wellbeing, equity, nature’s contribution to people |
| 22 | Shi H., Li X., Liu X., Wang S., Liu X., Zhang H., Tang D., Li T. | Global protected areas boost the carbon sequestration capacity: Evidences from econometric causal analysis | 2020 | Science of the Total Environment | SE interactions | Nature’s contribution to people |
| 23 | Estifanos T.K., Polyakov M., Pandit R., Hailu A., Burton M. | The impact of protected areas on the rural households’ incomes in Ethiopia | 2020 | Land Use Policy | Social | Human wellbeing |
| 24 | Poor E.E., Frimpong E., Imron M.A., Kelly M.J. | Protected area effectiveness in a sea of palm oil: A Sumatran case study | 2019 | Biological Conservation | Ecological | Forest cover |
| 25 | Rada S., Schweiger O., Harpke A., Kühn E., Kuras T., Settele J., Musche M. | Protected areas do not mitigate biodiversity declines: A case study on butterflies | 2019 | Diversity and Distributions | Ecological | Species richness |
| 26 | Geldmann J., Manica A., Burgess N.D., Coad L., Balmford A. | A global-level assessment of the effectiveness of protected areas at resisting anthropogenic pressures | 2019 | Proceedings of the National Academy of Sciences of the United States of America | SE interactions | Human pressures |
| 27 | Jones T., Hawes J.E., Norton G.W., Hawkins D.M. | Effect of protection status on mammal richness and abundance in Afromontane forests of the Udzungwa Mountains, Tanzania | 2019 | Biological Conservation | Ecological | Species population & richness |
| 28 | Brown J.A., Lockwood J.L., Avery J.D., Curtis Burkhalter J., Aagaard K., Fenn K.H. | Evaluating the long-term effectiveness of terrestrial protected areas: a 40-year look at forest bird diversity | 2019 | Biodiversity and Conservation | Ecological | Species population |
| 29 | Zhao H., Wu R., Long Y., Hu J., Yang F., Jin T., Wang J., Hu P., Wu W., Diao Y., Guo Y. | Individual-level performance of nature reserves in forest protection and the effects of management level and establishment age | 2019 | Biological Conservation | Ecological | Forest cover |
| 30 | Guadilla-Sáez S., Pardo-de-Santayana M., Reyes-García V., Svenning J.-C. | Biodiversity conservation effectiveness provided by a protection status in temperate forest commons of north Spain | 2019 | Forest Ecology and Management | Ecological | Species richness |
| 31 | Higginbottom T.P., Collar N.J., Symeonakis E., Marsden S.J. | Deforestation dynamics in an endemic-rich mountain system: Conservation successes and challenges in West Java 1990–2015 | 2019 | Biological Conservation | Ecological | Forest cover |
| 32 | Mansuy N., Miller C., Parisien M.-A., Parks S.A., Batllori E., Moritz M.A. | Contrasting human influences and macro-environmental factors on fire activity inside and outside protected areas of North America | 2019 | Environmental Research Letters | SE interactions | Human pressures |
| 33 | Miteva D.A., Ellis P.W., Ellis E.A., Griscom B.W. | The role of property rights in shaping the effectiveness of protected areas and resisting forest loss in the Yucatan Peninsula | 2019 | PLoS ONE | Ecological | Forest cover |
| 34 | Rosenblatt E., Creel S., Schuette P., Becker M.S., Christianson D., Dröge E., Mweetwa T., Mwape H., Merkle J., M'Soka J., Masonde J., Simpamba T. | Do protection gradients explain patterns in herbivore densities? An example with ungulates in Zambia's Luangwa valley | 2019 | PLoS ONE | Ecological | Species population |
| 35 | Achieng O., Richard M., Robert K., Nicholas O. | Effectiveness of Protected Area Governance in the Conservation of Mt. Marsabit Forest Ecosystem, Kenya | 2019 | Journal of Sustainable Forestry | SE interactions | Governance |
| 36 | Aditya V., Ganesh T. | Deciphering forest change: Linking satellite-based forest cover change and community perceptions in a threatened landscape in India | 2019 | Ambio | Ecological | Forest cover |
| 37 | Dähler N.B., Holderegger R., INFO FLORA, Bergamini A. | Effectiveness of Swiss protected areas in maintaining populations of rare vascular plants | 2019 | Journal for Nature Conservation | Ecological | Species population |
| 38 | Ma B., Cai Z., Zheng J., Wen Y. | Conservation, ecotourism, poverty, and income inequality – A case study of nature reserves in Qinling, China | 2019 | World Development | Social | Human wellbeing |
| 39 | Herrera D., Pfaff A., Robalino J. | Impacts of protected areas vary with the level of government: Comparing avoided deforestation across agencies in the Brazilian Amazon | 2019 | Proceedings of the National Academy of Sciences of the United States of America | Ecological | Forest cover |
| 40 | Naidoo R., Gerkey D., Hole D., Pfaff A., Ellis A.M., Golden C.D., Herrera D., Johnson K., Mulligan M., Ricketts T.H., Fisher B. | Evaluating the impacts of protected areas on human well-being across the developing world | 2019 | Science Advances | Social | Human wellbeing |
| 41 | Rodríguez-Rodríguez D., Martínez-Vega J. | Protected area effectiveness against land development in Spain | 2018 | Journal of Environmental Management | SE interactions | Human pressures |
| 42 | Li S., Wu J., Gong J., Li S. | Human footprint in Tibet: Assessing the spatial layout and effectiveness of nature reserves | 2018 | Science of the Total Environment | SE interactions | Human pressures |
| 43 | Hossain A.N.M., Lynam A.J., Ngoprasert D., Barlow A., Barlow C.G., Savini T. | Identifying landscape factors affecting tiger decline in the Bangladesh Sundarbans | 2018 | Global Ecology and Conservation | Ecological | Species population |
| 44 | Xavier da Silva M., Paviolo A., Tambosi L.R., Pardini R. | Effectiveness of Protected Areas for biodiversity conservation: Mammal occupancy patterns in the Iguaçu National Park, Brazil | 2018 | Journal for Nature Conservation | Ecological | Species population |
| 45 | Sarathchandra C., Dossa G.G.O., Ranjitkar N.B., Chen H., Deli Z., Ranjitkar S., de Silva K.H.W.L., Wickramasinghe S., Xu J., Harrison R.D. | Effectiveness of protected areas in preventing rubber expansion and deforestation in Xishuangbanna, Southwest China | 2018 | Land Degradation and Development | Ecological | Forest cover |
| 46 | López-Angarita J., Tilley A., Hawkins J.P., Pedraza C., Roberts C.M. | Land use patterns and influences of protected areas on mangroves of the eastern tropical Pacific | 2018 | Biological Conservation | Ecological | Forest cover |
| 47 | Assédé E.S.P., Djagoun C.A.M.S., Azihou F.A., Gogan Y.S.C., Kouton M.D., Adomou A.C., Geldenhuys C.J., Chirwa P.W., Sinsin B. | Efficiency of conservation areas to protect orchid species in Benin, West Africa | 2018 | South African Journal of Botany | Ecological | Species population |
| 48 | Duckworth G.D., Altwegg R. | Effectiveness of protected areas for bird conservation depends on guild | 2018 | Diversity and Distributions | Ecological | Species population |
| 49 | Mohseni F., Sabzghabaei G.R., Dashti S. | Identification of threat and pressure factors on protected areas using RAPPAM methodology (Case study: Khuzestan Province, Iran) | 2018 | Applied Ecology and Environmental Research | SE interactions | Human pressures |
| 50 | Jones N., Malesios C., Ioannidou E., Kanakaraki R., Kazoli F., Dimitrakopoulos P.G. | Understanding perceptions of the social impacts of protected areas: Evidence from three NATURA 2000 sites in Greece | 2018 | Environmental Impact Assessment Review | Social | Human wellbeing, equity |
| 51 | Muñoz Brenes C.L., Jones K.W., Schlesinger P., Robalino J., Vierling L. | The impact of protected area governance and management capacity on ecosystem function in Central America | 2018 | PLoS ONE | Ecological | Forest cover |
| 52 | Biró M., Bölöni J., Molnár Z. | Use of long-term data to evaluate loss and endangerment status of Natura 2000 habitats and effects of protected areas | 2018 | Conservation Biology | Ecological | Habitat extent |
| 53 | Canavire-Bacarreza G., Diaz-Gutierrez J.E., Hanauer M.M. | Unintended consequences of conservation: Estimating the impact of protected areas on violence in Colombia | 2018 | Journal of Environmental Economics and Management | Social | Human wellbeing |
| 54 | Beresford A.E., Buchanan G.M., Phalan B., Eshiamwata G.W., Balmford A., Brink A.B., Fishpool L.D.C., Donald P.F. | Correlates of long-term land-cover change and protected area performance at priority conservation sites in Africa | 2018 | Environmental Conservation | Ecological | Habitat extent |
| 55 | Tesfaw A.T., Pfaff A., Golden Kroner R.E., Qin S., Medeiros R., Mascia M.B. | Land-use and land-cover change shape the sustainability and impacts of protected areas | 2018 | Proceedings of the National Academy of Sciences of the United States of America | Ecological | Forest cover |
| 56 | Beauchamp E., Clements T., Milner-Gulland E.J. | Assessing Medium-term Impacts of Conservation Interventions on Local Livelihoods in Northern Cambodia | 2018 | World Development | Social | Human wellbeing |
| 57 | Kere E.N., Choumert J., Combes Motel P., Combes J.L., Santoni O., Schwartz S. | Addressing Contextual and Location Biases in the Assessment of Protected Areas Effectiveness on Deforestation in the Brazilian Amazônia | 2017 | Ecological Economics | Ecological | Forest cover |
| 58 | Butsic V., Munteanu C., Griffiths P., Knorn J., Radeloff V.C., Lieskovský J., Mueller D., Kuemmerle T. | The effect of protected areas on forest disturbance in the Carpathian Mountains 1985–2010 | 2017 | Conservation Biology | Ecological | Forest cover |
| 59 | Tapia-Armijos M.F., Homeier J., Draper Munt D. | Spatio-temporal analysis of the human footprint in South Ecuador: Influence of human pressure on ecosystems and effectiveness of protected areas | 2017 | Applied Geography | SE interactions | Human pressures |
| 60 | Blankespoor B., Dasgupta S., Wheeler D. | Protected areas and deforestation: new results from high-resolution panel data | 2017 | Natural Resources Forum | Ecological | Forest cover |
| 61 | García Márquez J.R., Krueger T., Páez C.A., Ruiz-Agudelo C.A., Bejarano P., Muto T., Arjona F. | Effectiveness of conservation areas for protecting biodiversity and ecosystem services: a multi-criteria approach | 2017 | International Journal of Biodiversity Science, Ecosystem Services and Management | Ecological & SE interactions | Species population & ecosystem services |
| 62 | Rosa-Velázquez M.I.D.L., Espinoza-Tenorio A., Díaz-Perera M.Á., Ortega-Argueta A., Ramos-Reyes R., Espejel I. | Development stressors are stronger than protected area management: A case of the Pantanos de Centla Biosphere Reserve, Mexico | 2017 | Land Use Policy | Ecological | Forest cover |
| 63 | Leon-Ortega M., Martinez J.E., Perez E., Lacalle J.A., Calvo J.F. | The contribution of non-protected areas to the conservation of Eurasian Eagle-owls in Mediterranean ecosystems | 2017 | Ecosphere | Ecological | Species population |
| 64 | Reddy C.S., Saranya K.R.L., Jha C.S., Dadhwal V.K., Murthy Y.V.N.K. | Earth observation data for habitat monitoring in protected areas of India | 2017 | Remote Sensing Applications: Society and Environment | Ecological | Forest cover |
| 65 | Li R., Xu M., Powers R., Zhao F., Jetz W., Wen H., Sheng Q. | Quantifying the evidence for co-benefits between species conservation and climate change mitigation in giant panda habitats | 2017 | Scientific Reports | SE interactions | Nature's contribution to People |
| 66 | Zhou C., Zhao Y., Connelly J.W., Li J., Xu J. | Current nature reserve management in China and effective conservation of threatened pheasant species | 2017 | Wildlife Biology | Ecological | Forest cover |
| 67 | Sudhakar Reddy C., Diwakar P.G., Krishna Murthy Y.V.N. | Sustainable Biodiversity Management in India: Remote Sensing Perspective | 2017 | Proceedings of the National Academy of Sciences India Section A - Physical Sciences | Ecological | Forest cover |
| 68 | Puhakka R., Pitkänen K., Siikamäki P. | The health and well-being impacts of protected areas in Finland | 2017 | Journal of Sustainable Tourism | Social | Human wellbeing |
| 69 | Duan W., Wen Y. | Impacts of protected areas on local livelihoods: Evidence of giant panda biosphere reserves in Sichuan Province, China | 2017 | Land Use Policy | Social | Human wellbeing |
| 70 | Pfaff A., Santiago-Ávila F., Joppa L. | Evolving protected-area impacts in Mexico: Political shifts as suggested by impact evaluations | 2017 | Forests | Ecological | Forest cover |
| 71 | Eklund J., Blanchet F.G., Nyman J., Rocha R., Virtanen T., Cabeza M. | Contrasting spatial and temporal trends of protected area effectiveness in mitigating deforestation in Madagascar | 2016 | Biological Conservation | Ecological | Forest cover |
| 72 | Hiley J.R., Bradbury R.B., Thomas C.D. | Impacts of habitat change and protected areas on alpha and beta diversity of Mexican birds | 2016 | Diversity and Distributions | Ecological | Species population & richness |
| 73 | Zhang Y., Hu Z., Qi W., Wu X., Bai W., Li L., Ding M., Liu L., Wang Z., Zheng D. | Assessment of effectiveness of nature reserves on the Tibetan Plateau based on net primary production and the large sample comparison method | 2016 | Journal of Geographical Sciences | Ecological | Net primary production |
| 74 | Zorrilla-Pujana J., Rossi S. | Environmental education indicators system for protected areas management | 2016 | Ecological Indicators | Social | Education |
| 75 | Kisingo A., Rollins R., Murray G., Dearden P., Clarke M. | Evaluating ‘good governance’: The development of a quantitative tool in the Greater Serengeti Ecosystem | 2016 | Journal of Environmental Management | Social | Equity |
| 76 | Yu B., Chao X., Zhang J., Xu W., Ouyang Z. | Effectiveness of nature reserves for natural forests protection in tropical Hainan: a 20 year analysis | 2016 | Chinese Geographical Science | Ecological | Forest cover |
| 77 | Butsic V., Baumann M., Shortland A., Walker S., Kuemmerle T. | Conservation and conflict in the Democratic Republic of Congo: The impacts of warfare, mining, and protected areas on deforestation | 2015 | Biological Conservation | Ecological | Forest cover |
| 78 | Wendland K.J., Baumann M., Lewis, Sieber A., Radeloff V.C. | Protected area effectiveness in European Russia: A postmatching panel data analysis | 2015 | Land Economics | Ecological | Forest cover |
| 79 | Françoso R.D., Brandão R., Nogueira C.C., Salmona Y.B., Machado R.B., Colli G.R. | Habitat loss and the effectiveness of protected areas in the Cerrado Biodiversity Hotspot | 2015 | Natureza e Conservacao | Ecological | Forest cover |
| 80 | Brandt J.S., Butsic V., Schwab B., Kuemmerle T., Radeloff V.C. | The relative effectiveness of protected areas, a logging ban, and sacred areas for old-growth forest protection in southwest China | 2015 | Biological Conservation | Ecological | Forest cover |
| 81 | Brun C., Cook A.R., Lee J.S.H., Wich S.A., Koh L.P., Carrasco L.R. | Analysis of deforestation and protected area effectiveness in Indonesia: A comparison of Bayesian spatial models | 2015 | Global Environmental Change | Ecological | Forest cover |
| 82 | Barnes M., Szabo J.K., Morris W.K., Possingham H. | Evaluating protected area effectiveness using bird lists in the Australian Wet Tropics | 2015 | Diversity and Distributions | Ecological | Species population |
| 83 | Hanauer M.M., Canavire-Bacarreza G. | Implications of heterogeneous impacts of protected areas on deforestation and poverty | 2015 | Philosophical Transactions of the Royal Society B: Biological Sciences | Ecological & Social | Forest cover & poverty |
| 84 | Miteva D.A., Murray B.C., Pattanayak S.K. | Do protected areas reduce blue carbon emissions? A quasi-experimental evaluation of mangroves in Indonesia | 2015 | Ecological Economics | Ecological | Forest cover |
| 85 | González-Maya J.F., Víquez-R L.R., Belant J.L., Ceballos G. | Effectiveness of protected areas for representing species and populations of terrestrial mammals in Costa Rica | 2015 | PLoS ONE | Ecological | Species population & richness |
| 86 | Paiva R.J.O., Brites R.S., Machado R.B. | The role of protected areas in the avoidance of anthropogenic conversion in a high pressure region: A matching method analysis in the core region of the Brazilian cerrado | 2015 | PLoS ONE | Ecological | Forest cover |
| 87 | Shah P., Baylis K. | Evaluating heterogeneous conservation effects of forest protection in Indonesia | 2015 | PLoS ONE | Ecological | Forest cover |
| 88 | Bragina E.V., Radeloff V.C., Baumann M., Wendland K., Kuemmerle T., Pidgeon A.M. | Effectiveness of protected areas in the Western Caucasus before and after the transition to post-socialism | 2015 | Biological Conservation | Ecological | Forest cover |
| 89 | Diniz M.F., Brito D. | Protected areas effectiveness in maintaining viable giant anteater (*Myrmecophaga tridactyla*) populations in an agricultural frontier | 2015 | Natureza e Conservacao | Ecological | Species population |
| 90 | Mayrhofer S., Kirchmeir H., Weigand E., Mayrhofer M. | Assessment of forest wilderness in Kalkalpen National Park | 2015 | Eco.mont | Ecological | Species population |
| 91 | Furlonge T., Dyer F., Davis J. | The influence of differing protected area status and environmental factors on the macroinvertebrate fauna of temperate austral wetlands | 2015 | Global Ecology and Conservation | Ecological | Species richness |
| 92 | Pfaff A., Robalino J., Herrera D., Sandoval C. | Protected areas?impacts on Brazilian Amazon deforestation: Examining conservation - Development interactions to inform planning | 2015 | PLoS ONE | Ecological | Forest cover |
| 93 | Bonet-García F.J., Pérez-Luque A.J., Moreno-Llorca R.A., Pérez-Pérez R., Puerta-Piñero C., Zamora Rodríguez R.J. | Protected areas as elicitors of human well-being in a developed region: A new synthetic (socioeconomic) approach | 2015 | Biological Conservation | Social | Human wellbeing |
| 94 | Ferraro P.J., Hanauer M.M., Miteva D.A., Nelson J.L., Pattanayak S.K., Nolte C., Sims K.R.E. | Estimating the impacts of conservation on ecosystem services and poverty by integrating modeling and evaluation | 2015 | Proceedings of the National Academy of Sciences of the United States of America | Social & SE interactions | Nature’s contribution to people, human wellbeing |
| 95 | Rayner L., Lindenmayer D.B., Wood J.T., Gibbons P., Manning A.D. | Are protected areas maintaining bird diversity? | 2014 | Ecography | Ecological | Forest cover & species richness |
| 96 | Scolozzi R., Schirpke U., Morri E., D'Amato D., Santolini R. | Ecosystem services-based SWOT analysis of protected areas for conservation strategies | 2014 | Journal of Environmental Management | SE interactions | Ecosystem services |
| 97 | Rosa I.M.D., Purves D., Carreiras J.M.B., Ewers R.M. | Modelling land cover change in the Brazilian Amazon: temporal changes in drivers and calibration issues | 2014 | Regional Environmental Change | Ecological | Forest cover |
| 98 | Shivaprakash K.N., Ramesha B.T., Shaanker R.U., Dayanandan S., Ravikanth G. | Genetic structure, diversity and long term viability of a medicinal plant, *nothapodytes nimmoniana graham*. (Icacinaceae), in protected and non-protected areas in the Western Ghats biodiversity hotspot | 2014 | PLoS ONE | Ecological | Species population |
| 99 | Carranza T., Balmford A., Kapos V., Manica A. | Protected area effectiveness in reducing conversion in a rapidly vanishing ecosystem: The Brazilian Cerrado | 2014 | Conservation Letters | Ecological | Forest cover |
| 100 | Clements T., Suon S., Wilkie D.S., Milner-Gulland E.J. | Impacts of Protected Areas on Local Livelihoods in Cambodia | 2014 | World Development | Social & SE interactions | Human wellbeing, nature’s contribution to people |
| 101 | Hall J.M., Burgess N.D., Rantala S., Vihemäki H., Jambiya G., Gereau R.E., Makonda F., Njilima F., Sumbi P., Kizaji A. | Ecological and Social Outcomes of a New Protected Area in Tanzania | 2014 | Conservation Biology | Ecological & social | Forest cover, human wellbeing |
| 102 | Haruna A., Pfaff A., Van Den Ende S., Joppa L. | Evolving protected-area impacts in Panama: Impact shifts show that plans require anticipation | 2014 | Environmental Research Letters | Ecological | Forest cover |
| 103 | Green J.M.H., Larrosa C., Burgess N.D., Balmford A., Johnston A., Mbilinyi B.P., Platts P.J., Coad L. | Deforestation in an African biodiversity hotspot: Extent, variation and the effectiveness of protected areas | 2013 | Biological Conservation | Ecological | Forest cover |
| 104 | Sieber A., Kuemmerle T., Prishchepov A.V., Wendland K.J., Baumann M., Radeloff V.C., Baskin L.M., Hostert P. | Landsat-based mapping of post-Soviet land-use change to assess the effectiveness of the Oksky and Mordovsky protected areas in European Russia | 2013 | Remote Sensing of Environment | Ecological | Forest cover |
| 105 | Nolte C., Agrawal A. | Linking Management Effectiveness Indicators to Observed Effects of Protected Areas on Fire Occurrence in the Amazon Rainforest | 2013 | Conservation Biology | SE interactions | Human pressures |
| 106 | Durán A.P., Casalegno S., Marquet P.A., Gaston K.J. | Representation of ecosystem services by terrestrial protected areas: Chile as a case study | 2013 | PLoS ONE | Ecological & SE interactions | Habitat quality, sensitive species richness & ecosystem services |
| 107 | Wang W., Pechacek P., Zhang M., Xiao N., Zhu J., Li J. | Effectiveness of Nature Reserve System for Conserving Tropical Forests: A Statistical Evaluation of Hainan Island, China | 2013 | PLoS ONE | Ecological | Forest cover |
| 108 | Rodríguez N., Armenteras D., Retana J. | Effectiveness of protected areas in the Colombian Andes: Deforestation, fire and land-use changes | 2013 | Regional Environmental Change | Ecological | Forest cover |
| 109 | Beresford A.E., Eshiamwata G.W., Donald P.F., Balmford A., Bertzky B., Brink A.B., Fishpool L.D.C., Mayaux P., Phalan B., Simonetti D., Buchanan G.M. | Protection Reduces Loss of Natural Land-Cover at Sites of Conservation Importance across Africa | 2013 | PLoS ONE | Ecological | Forest cover |
| 110 | Gimmi U., Radeloff V.C. | Assessing naturalness in northern great lakes forests based on historical land-cover and vegetation changes | 2013 | Environmental Management | Ecological | Forest cover |
| 111 | Menconi M.E., Grohmann D. | Statistical assessment of vegetation dynamics within protected areas using remote sensing data | 2013 | Journal of Agricultural Engineering | Ecological | Forest cover |
| 112 | Andam K.S., Ferraro P.J., Hanauer M.M. | The effects of protected area systems on ecosystem restoration: A quasi-experimental design to estimate the impact of Costa Rica's protected area system on forest regrowth | 2013 | Conservation Letters | Ecological | Forest cover |
| 113 | Knorn J., Kuemmerle T., Radeloff V.C., Szabo A., Mindrescu M., Keeton W.S., Abrudan I., Griffiths P., Gancz V., Hostert P. | Forest restitution and protected area effectiveness in post-socialist Romania | 2012 | Biological Conservation | Ecological | Forest cover |
| 114 | Vuohelainen A.J., Coad L., Marthews T.R., Malhi Y., Killeen T.J. | The effectiveness of contrasting protected areas in preventing deforestation in Madre de Dios, Peru | 2012 | Environmental Management | Ecological | Forest cover |
| 115 | Lu D.-J., Kao C.-W., Chao C.-L. | Evaluating the management effectiveness of five protected areas in Taiwan using WWF's RAPPAM | 2012 | Environmental Management | SE interactions | Human pressures |
| 116 | Gaveau D.L.A., Curran L.M., Paoli G.D., Carlson K.M., Wells P., Besse-Rimba A., Ratnasari D., Leader-Williams N. | Examining protected area effectiveness in Sumatra: Importance of regulations governing unprotected lands | 2012 | Conservation Letters | Ecological | Forest cover |
| 117 | Dávalos L.M., Bejarano A.C., Hall M.A., Correa H.L., Corthals A., Espejo O.J. | Forests and drugs: Coca-driven deforestation in tropical biodiversity hotspots | 2011 | Environmental Science and Technology | Ecological | Forest cover |
| 118 | Ferraro P.J., Hanauer M.M. | Protecting ecosystems and alleviating poverty with parks and reserves: 'Win-win' or tradeoffs? | 2011 | Environmental and Resource Economics | Ecological & Social | Forest cover & poverty |
| 119 | Tang Z., Fang J., Sun J., Gaston K.J. | Effectiveness of protected areas in maintaining plant production | 2011 | PLoS ONE | Ecological | Forest cover |
| 120 | Klorvuttimontara S., McClean C.J., Hill J.K. | Evaluating the effectiveness of protected areas for conserving tropical forest butterflies of Thailand | 2011 | Biological Conservation | Ecological | Forest cover & species richness |
| 121 | Greve M., Chown S.L., van Rensburg B.J., Dallimer M., Gaston K.J. | The ecological effectiveness of protected areas: A case study for South African birds | 2011 | Animal Conservation | Ecological | Species population & richness |
| 122 | Kurdoǧlu O., Çokçalişkan B.A. | Assessing the effectiveness of protected area management in the Turkish Caucasus | 2011 | African Journal of Biotechnology | SE interactions | Human pressures |
| 123 | Houehanou T.D., Kindomihou V., Sinsin B. | Effectiveness of conservation areas in protecting Shea trees against hemiparasitic plants (Loranthaceae) in Benin, West Africa | 2011 | Plant Ecology and Evolution | Ecological | Diseases |
| 124 | Helsen K., van Meerbeek K., Honnay O., Hermy M. | Conservation credit for plant species diversity of small nature reserves in an agricultural matrix | 2011 | Plant Ecology and Evolution | Ecological | Species richness |
| 125 | Rayn D., Sutherland W.J. | Impact of nature reserve establishment on deforestation: A test | 2011 | Biodiversity and Conservation | Ecological | Forest cover |
| 126 | Joppa L.N., Pfaff A. | Global protected area impacts | 2011 | Proceedings of the Royal Society B: Biological Sciences | Ecological | Forest cover |
| 127 | Radeloff V.C., Stewart S.I., Hawbaker T.J., Gimmi U., Pidgeon A.M., Flather C.H., Hammer R.B., Helmers D.P. | Housing growth in and near United States protected areas limits their conservation value | 2010 | Proceedings of the National Academy of Sciences of the United States of America | SE interactions | Human pressures |
| 128 | Wanyama F., Muhabwe R., Plumptre A.J., Chapman C.A., Rothman J.M. | Censusing large mammals in Kibale National Park: Evaluation of the intensity of sampling required to determine change | 2010 | African Journal of Ecology | Ecological | Species population |
| 129 | Matar D.A., Anthony B.P. | Application of modified threat reduction assessments in Lebanon | 2010 | Conservation Biology | SE interactions | Human pressures |
| 130 | Gandariasbeitia I.E. | Socioeconomic impacts of national parks: A case study from the north-east of England | 2010 | Lurralde: Investigacion y Espacio | Social & SE interactions | Human wellbeing & nature's contribution to People |
| 131 | Andam K.S., Ferraro P.J., Sims K.R.E., Healy A., Holland M.B. | Protected areas reduced poverty in Costa Rica and Thailand | 2010 | Proceedings of the National Academy of Sciences of the United States of America | Social | Human wellbeing |
| 132 | Htun N.Z., Mizoue N., Kajisa T., Yoshida S. | Deforestation and forest degradation as measures of Popa Mountain Park (Myanmar) effectiveness | 2009 | Environmental Conservation | Ecological | Forest cover |
| 133 | Brown K.A., Carter Ingram J., Flynn D.F.B., Razafindrazaka R., Jeannoda V. | Protected area safeguard tree and shrub communities from degradation and invasion: A case study in eastern Madagascar | 2009 | Environmental Management | Ecological | Species richness |
| 134 | Andam K.S., Ferraro P.J., Pfaff A., Sanchez-Azofeifa G.A., Robalino J.A. | Measuring the effectiveness of protected area networks in reducing deforestation | 2008 | Proceedings of the National Academy of Sciences of the United States of America | Ecological | Forest cover |
| 135 | Nagendra H. | Do parks work? Impact of protected areas on land cover clearing | 2008 | Ambio | Ecological | Habitat extent |
| 136 | De Sherbinin A. | Is poverty more acute near parks? An assessment of infant mortality rates around protected areas in developing countries | 2008 | ORYX | Social | Human wellbeing |
| 137 | Kuemmerle T., Hostert P., Radeloff V.C., Perzanowski K., Kruhlov I. | Post-socialist forest disturbance in the Carpathian border region of Poland, Slovakia, and Ukraine | 2007 | Ecological Applications | Ecological | Forest cover |
| 138 | Hayes T.M. | Parks, People, and Forest Protection: An Institutional Assessment of the Effectiveness of Protected Areas | 2006 | World Development | Ecological | Forest cover |
| 139 | Román-Cuesta R.M., Martínez-Vilalta J. | Effectiveness of protected areas in mitigating fire within their boundaries: Case study of Chiapas, Mexico | 2006 | Conservation Biology | SE interactions | Human pressures |
| 140 | Bleher B., Uster D., Bergsdorf T. | Assessment of threat status and management effectiveness in Kakamega Forest, Kenya | 2006 | Biodiversity and Conservation | Ecological | Forest cover |
| 141 | Saayman M., Saayman A. | Creating a framework to determine the socio-economic impact of national parks in South Africa: A case study of the Addo Elephant National Park | 2006 | Tourism Economics | Social | Human wellbeing |
| 142 | Mas J.-F. | Assessing protected area effectiveness using surrounding (buffer) areas environmentally similar to the target area | 2005 | Environmental Monitoring and Assessment | Ecological | Forest cover |
| 143 | Korhonen K., Rahkonen O., Hemminki E. | Implications of integrated nature conservation for human reproductive health: A case study from Ranomafana National Park, Madagascar | 2004 | Development Southern Africa | Social | Human wellbeing |
| 144 | Getzner M. | The economic impact of national parks: The perception of key actors in Austrian national parks | 2003 | International Journal of Sustainable Development | Social | Equity |
| 145 | Walpole M.J., Goodwin H.J. | Local attitudes towards conservation and tourism around Komodo National Park, Indonesia | 2001 | Environmental Conservation | Social | Equity, human wellbeing |
| 146 | Fortin M.-J., Gagnon C. | An assessment of social impacts of national parks on communities in Quebec, Canada | 1999 | Environmental Conservation | Social | Equity, human wellbeing |
| 147 | Nepal S.K. | Sustainable tourism, protected areas and livelihood needs of local communities in developing countries | 1997 | International Journal of Sustainable Development and World Ecology | Social | Equity, human wellbeing |
| 148 | Bennett J., Gillespie R., Powell R., Chalmers L. | The economic value and regional economic impact of national parks | 1996 | Australian Journal of Environmental Management | Social | Human wellbeing |
| 149 | Place S.E.A. | The Impact of National Park Development on Tortuguero, Costa Rica | 1988 | Journal of Cultural Geography | Social | Human wellbeing |
| 150 | [No author name available] | National Parks: a study of rural economies ( UK). | 1981 | UK Countryside Commission Publication | Social | Human wellbeing |
